# Organism-like formation of Schistosoma hemozoin and its function suggest a mechanism for anti-malarial action of artemisinin

**DOI:** 10.1038/srep34463

**Published:** 2016-10-03

**Authors:** Jun Sun, Chen Li, Suwen Wang

**Affiliations:** 1 Institute for Infectious Diseases and Vaccine Development, Tongji University School of Medicine, 1239 Siping Road, Shanghai 200092, P.R. China

## Abstract

The current theories of antimalarial mechanism of artemisinin are inadequate to fully explain the observed effects. In our study, “organism-like” formation of *Schistosoma* hemozoin granules by attaching to and utilizing erythrocytes to form new ones was observed. This indicates that heme iron is transferred from erythrocytes to hemozoin granules during their formation. However, as a disposal product of heme detoxification, these granules are not completely expelled from the *Schistosoma* gut, but decomposed again between microvilli in the posterior portion of the gut to transfer iron to eggs. Based on the function of iron transport supported by our observation of the unique process of *Schistosoma* hemozoin formation, here we propose a new viewpoint of antimalarial mechanism of artemisinin, which emphasizes the final outcome, i.e., interference of iron utilization in parasites by artemisinin, instead of focusing on the mode of interaction between artemisinin and heme or hemozoin. This suggests that artemisinin and its endoperoxides derivatives likely hit the Achilles’ heel of hemozoin-producing and iron-dependent organisms.

Artemisinin and its endoperoxides derivatives are potent drugs well known for their ability to kill malaria parasites in the blood of patients. In recent years, resistance of malaria parasites to artemisinin, which first emerged in Cambodia, has spread over the mainland of Southeast Asia^1^,^2^. This has posed a serious threat of untreatable malaria^3^. Thus, elucidation of the antimalarial mechanism of artemisinin is urgently needed in order to promote research and development of new antimalarial drugs. Malaria parasites are hemozoin-producing organisms. Its hemozoin has been gradually considered an active “inert” substance^4^ and attractive target for developing drugs to treat malaria. Many popular antimalarial drugs, such as chloroquine and mefloquine, are capable of killing malaria parasites by inhibiting hemozoin formation. Obviously, hemozoin is essential to the survival of malaria parasites. Research into hemozoin targeting has been hindered by difficulties in investigating the biological functions and characteristics of hemozoin formation in a single cell. Fortunately, Schistosomes, which are multicellular hemozoin-producing organisms, offer the possibility to understand the significance of hemozoin formation. In particular, artemisinin can interact with heme or hemozoin and specifically kill the two hemozoin-producing organisms, suggesting that similar mechanisms, associated with heme or hemozoin, could be utilized by artemisinin. Although the interaction between hemozoin and artemisinin has been researched, the relationship between the significance of hemozoin formation and antimalarial mechanism of artemisinin remains to be investigated.

Some researches propose that artemisinin or its derivatives can act on some target proteins to kill malarial parasites, such as cysteine protease^5^, *Plasmodium falciparum* exported protein 1 (PfEXP1)^6^, *P. falciparum* calcium ATPase 6(PfATP6)^7^, *P. falciparum* translationally controlled tumour protein (PfTCTP)^8^, Pfmdr1^9^, K13-propeller protein^10^,^11^, phosphatidylinositol-3-kinase (PfPI3K)^12^ and so on. However, it is inconceivable that artemisinin or its distinctly variable derivatives with personalized configuration of the atoms, except for their sharing of the endoperoxidic bridge, can act on the same single protein in malarial parasites. Electron microscopic evidences have revealed that the digestive vacuole membrane suffers damage soon after malarial parasites are exposed to artemisinin^13^, whereas fluorescently tagged artemisinin have been seen in the Golgi, ER and mitochondria^14^. Moreover, mitochondria can be damaged by artemisinin^15^. Discrepant observations by different research groups make this problem very controversial. Although hypotheses such as the reactive species hypothesis involving oxygen and C-centred radicals or carbocations, and the heme (Fe) activation hypothesis (or heme model) are related, they are also different from each other. At present, there is no decisive evidence pointing towards the absolute validity of any of the proposed theories relating to a direct effect on the parasite. The heme model, which is one of the major theories, has received wide acceptance to explain the mechanism of action of artemisinin. In this model, heme from haemoglobin degradation interacts with artemisinin. Iron in the heme directly reduces the peroxide bond in artemisinin, thereby generating reactive oxygen radicals that damage the parasite, thereby leading to its death^16^. However, evidences from some studies do not support this model. For example, C-centered radicals are too short-lived to favor intermolecular interactions^17^. Furthermore, the parasiticidal effects of artemisinin on early ring-stage malaria parasites with little heme or hemozoin, also argue against heme-dependent activation theory^18^. These observations weaken support to the heme model. On the other hand, they suggest that previous models may be missing a key part of the antimalarial mechanism of artemisinin.

Although there is no consensus about the mechanism of action through which artemisinin kills malaria parasites, several lines of evidence show that artemisinin exerts antimalarial action by radical formation which depends on the endoperoxide bridge. During the process, the interaction of iron in heme with the endoperoxide bridge has been confirmed to generate reactive oxygen radicals. Most researchers have paid much more attention to the radicals and their damages in mechanism of action of artemisinin, but not on the fact that heme is consumed by artemisinin and hemozoin formation is interfered. Since hemozoin is a disposal product formed during detoxification of free heme, it should be removed from malarial parasites or *Schistosoma* guts in a timely manner. However, hemozoin has always been found to accumulate in malarial parasites or worm guts. Furthermore, hemozoin nearly accompanies their overall mature stages, like a cell “organ” in the malarial parasite cell. Why is it not excluded completely from the cell? It is not easy to investigate the characteristics and rules of hemozoin formation within a malarial parasite. Moreover, the frequently-studied synthetic analogs of malarial hemozoin, such as β-hematin, do not easily reveal hemozoin-related biological processes either. However, *Schistosoma* hemozoin, which is very similar to malarial hemozoin, is formed in the gut and can be observed *in situ* or conveniently obtained for in-depth analyse of the characteristics and potential function of hemozoin. In fact, unique process of hemozoin formation suggests its unexpected role *in vivo*, which can improve our understanding of the formation and potential role of malarial hemozoin.

## Results and Discussion

### Morphological characteristics of *Schistosoma* hemozoin granules

**Figure 1:**
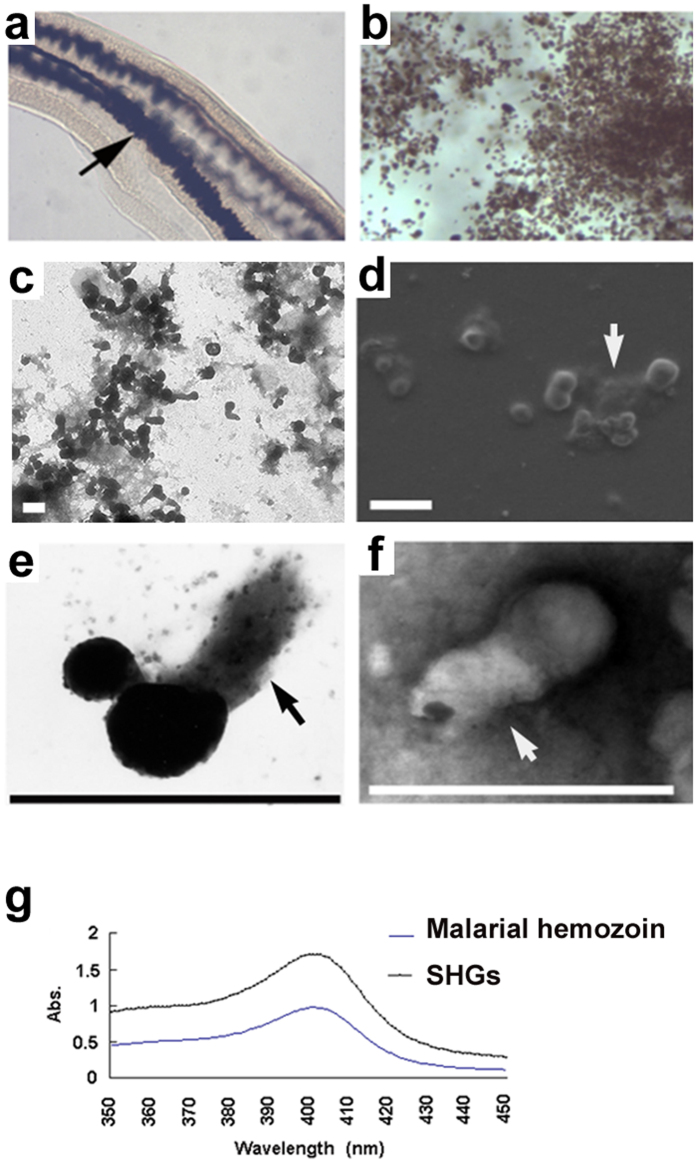
Morphology of *S. japonicum* hemozoin granules (SHGs). (**a**) *S. japonicum* gut is filled with SHGs, as observed under light microscope (LM) (100×) (arrow). (**b**) SHGs under LM (1000×). (**c**) The TEM image shows globe- and comma-shaped granules (SHGs) attaching to the cell debris. (**d**) The ESEM image shows SHGs attached to the plate with their amorphous tails and (**e**) The SHG with its amorphous tail under TEM. (**f**) The TEM image showed a negatively stained SHG with 1% phosphotungstic acid. The head of the comma-shaped granule is already fully formed, whereas the tail is still amorphous. (**g**) SHGs showed the same absorption peak at 400 nm as malarial hemozoin. All scale bars indicate 1 μm.

In this study, the morphological characteristics of Schistosoma hemozoin granules (SHGs) were observed in different ways to analyze hemozoin formation. SHGs from the gut contents of females showed a characteristic dark brown colour under light microscopy (LM) (Fig. 1a,b), which served as a natural and effective identification mark. Under transmission electron microscopy (TEM), these electron-dense granules appeared to be globular or comma-shaped, with tail lengths varying from 0.2 μm to 1 μm (Fig. 1c and Supplementary Fig. 1). Field emission scanning electron microscopy (FESEM) and TEM showed that electron density in the SHG tail was different from that in its head (Fig. 1d–f). Moreover, in the presence of SDS, the tail of hemozoin granule is easily dissolved (Supplementary Fig. 2), indicating that SHG is a complex of hemozoin and lipids. In addition, SHGs shared the same absorption peak at 400 nm with malarial hemozoin (Fig. 1g), suggesting that they have similar chemical compositions. Since SHGs are crystals of heme, they are undoubtedly iron-containing granules, which was confirmed in our previous study^19^.

**Figure 2:**
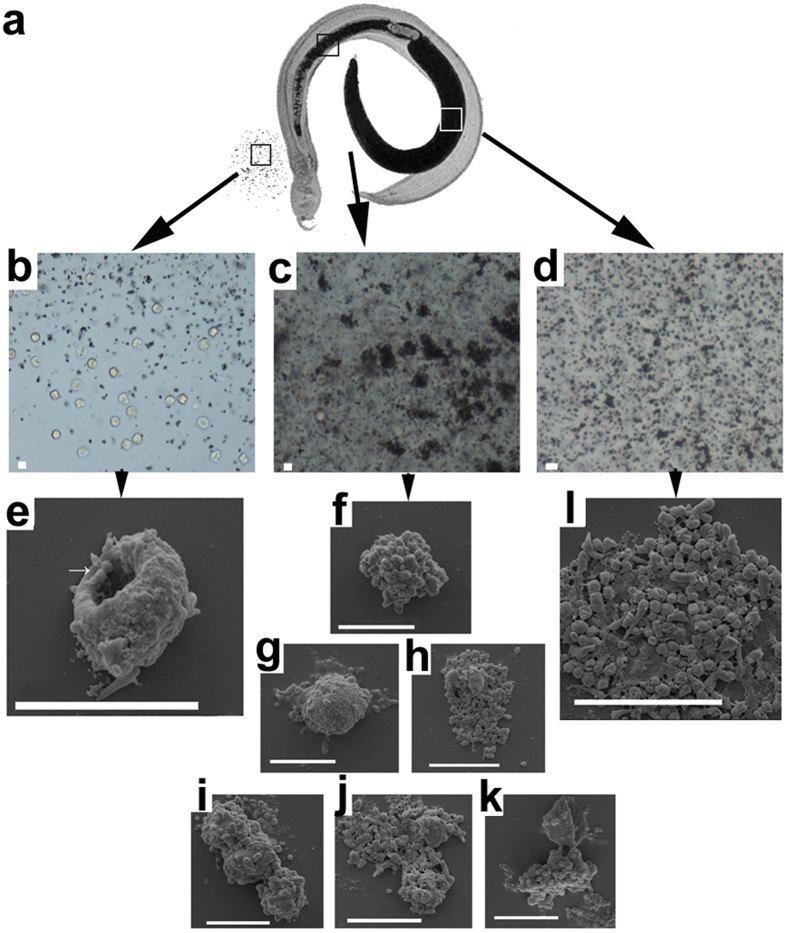
Interaction of *S. japonicum* hemozoin granules (SHGs) and erythrocytes in *S. japonicum* gut. (**a**) A pair of *S. japonicum*. The female contained dark brown SHGs in its gut. (**b**) Under LM, the erythrocytes and SHGs were released from the mouth of female worm. (**c**) In the middle of the gut, the erythrocytes were attached and ‘surrounded’ by SHGs under LM. (**d**) In the posterior portion of the gut, only free SHGs and SHG clusters were observed under LM. (**e**) FESEM image shows comma-shaped SHGs attached to erythrocytes with a biconcave disc shape in the vomitus from the mouth of female worms. (**f**–**k**) Erythrocytes still retained their size, but typical morphology disappeared. Some erythrocytes were replaced by clusters of globular and comma-shaped SHGs with an erythrocyte outline under FEG-ESEM. (**l**) SHGs in the posterior portion of the gut under FESEM. All scale bars indicate 5 μm.

To investigate the interaction between erythrocytes and hemozoin granules, we observed erythrocytes and hemozoin granules in different portions of the digestive tract of *S. japonicum* with various methods (Fig. 2). Spit from the mouth of female worms showed that the recently-ingested erythrocytes retained their typical biconcave disc shapes (Fig. 2b). FESEM images revealed that some erythrocytes were “punctuated” with SHGs (Fig. 2e), revealing the original interaction between erythrocytes and SHGs. In the gut section between the mouth and the ovary, some erythrocytes lost their typical morphology and were ‘wrapped’ by dark brown SHGs (Fig. 2c). FESEM showed that SHGs with various sizes ‘embedded’ themselves in the damaged erythrocytes (Fig. 2f–k). Moreover, some ‘wrapped erythrocytes’ disappeared and left a group of SHGs with the outline of an erythrocyte (Fig. 2h,j,k). By contrast, the gut section after the ovary and adjacent to the vitelline glands only contained individual dark brown granules or clusters of granules (Fig. 2d,l). Based on the abovementioned observations, the issue is whether the SHGs destroy erythrocytes only by attaching to them or that erythrocytes are attached to and utilized by SGHs to form new SHGs.

### Interaction between SHGs and erythrocytes

**Figure 3:**
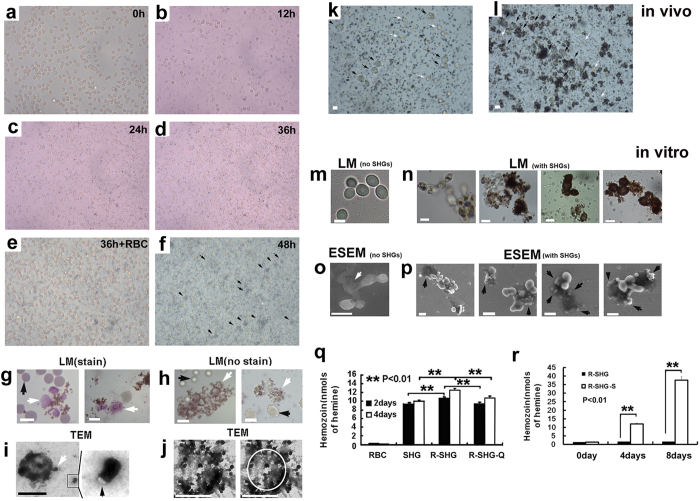
Interaction of SHGs and cells *in vitro*. (**a**–**f**) SHGs (10 nM) and erythrocytes (10^3–4 ^cells/ml) were mixed and cultured. (**a**) At 0 h; (**b**) At 12 h, most erythrocytes were attached by dark brown SHGs and lysed; (**c**) At 24 h; (**d**) At 36h, more SHGs appeared. (**e**) New erythrocytes were complemented to the culture. (**f**) At 48 h, more SHGs appeared. (**g**) Dark brown SHGs attached to and damaged erythrocytes. After giemsa’s stain, the damaged ones showed different colour and shapes. Normal erythrocytes (black arrow); Damaged erythrocytes (white arrow). (**h**) Without giemsa’s stain, SHGs attached to and destroyed erythrocytes, finally formed a SHGs circle around the erythrocytes. Normal ones (black arrow); Damaged ones (white arrow). (i) A comma-shaped granule (white arrow) attached to an erythrocyte with its lipid-like tail (black arrow). (**j**) An erythrocyte was degraded, accompanied with the formation of SHGs along the cell membrane. The white circle indicates the outline of an erythrocyte. (**k**,**l**) Aside from erythrocytes (white arrows), SHGs also attached to and destroyed other cells, as detected in the *S. japonicum* gut (black arrows). (**m**) *Saccharomyces* cells. (**n**) After *Saccharomyces* cells, SHGs and erythrocyte supernatant were mixed and incubated for 4–8 days, SHGs attached and leaded to the degradation of *Saccharomyces* cells and the formation of new SHGs in the debris. (**o**) *Saccharomyces* cells and its debris. No granules were detected in the debris under ESEM (white arrow). (**p**) *Saccharomyces* cells were degraded and new SHGs occurred from the debris (black arrows). (**q**) In the mixture of erythrocytes and SHGs, the amount of SHGs significantly increased in chloroquine-sensitive reactions. (**r**) In the mixture of erythrocytes or supernatant of broken erythrocytes, SHGs and *Saccharomycetes*, the amount of SHGs significantly increased than that in the mixture of erythrocytes and SHGs. RBC: erythrocytes; SHG: *S. japonicum* hemozoin granules; R-SHG: the mixture of erythrocytes and SHGs; R-SHG-Q: the mixture of erythrocytes, SHGs and chloroquine; R-SHG-S: the mixture of erythrocytes, SHGs and *Saccharomycetes*. The plot shows the mean ± standard error of the mean (SEM). All scale bars, 5 μm.

To reveal the interaction between SHGs and erythrocytes, we incubated a mixture of erythrocytes and individual SHGs *in vitro* (Fig. 3a). After 12 h of incubation, we found that SHGs attached to nearly all erythrocytes (Fig. 3b), confirming that SHGs are able to attach to the cells. After 24 h of incubation, tiny SHGs gradually appeared at the surface of erythrocytes or at the bottom of culture plates, and their colour turned browner after 36 h of incubation (Fig. 3c,d). If the mixture did not contain sufficient erythrocytes or heme, SHGs formed slowly or stopped forming. However, after more erythrocytes or supernatant of broken erythrocytes (SBE; containing heme), rather than SHGs, were added, we observed more brown SHGs forming around the degrading erythrocytes (Fig. 3e,f), as in the *Schistosoma* gut. This process can be clearly observed in Supplementary video file 1. It showed that in the culture, erythrocytes were degraded after SHGs attached to the erythrocytes (Fig. 3g–i), and then new SHG granules were observed to form gradually along the cell membrane (Fig. 3h–j). Simultaneously, we analysed the quantity of SHG in the mixture. We found that the quantity of SHGs significantly increased from 9.3 nmol to 10.7 nmol and from 9.9 nmol to 12.5 nmol after 2 and 4 days of incubation in chloroquine-sensitive reactions, respectively (Fig. 3q). We could even observe change in erythrocyte debris from a group of colourless debris with a few dark brown SHGs to a group with increasing number of dark brown SHGs, which marked the gradual process of utilization of erythrocyte debris to form more new SHGs (Supplementary Fig. 3). The abovementioned observation strongly confirmed that the increase of SHGs depended on *de novo* assembly of free heme released from erythrocytes, rather than aggregation of pre-existing SHGs. Notably, in the culture, only the erythrocyte attached by SHGs can be utilized to form new ones by self-assembly. Malarial hemozoin has also been reported to self-assemble, depending on availability of pre-existing hemozoin^20^. This suggests that SHGs and malarial hemozoin form via similar mechanisms.

We then asked how SHGs become globe- or comma-shaped granules. To reveal this, we observed a series of changes of SHGs. We found that erythrocytes were degraded to debris, based on which globe-shaped SHGs assembled first. Then, the components of debris aggregated to the tail, which resulted in a comma-shaped granule. Supplementary Fig. 4 shows a process of formation from heme microcrystalline in erythrocyte debris to a comma-shaped granule. Thus, the formation of a globe- or comma-shaped SHG granule depends on the electron-dense part of erythrocyte debris.

### Morphological characteristics of malarial hemozoin

**Figure 4:**
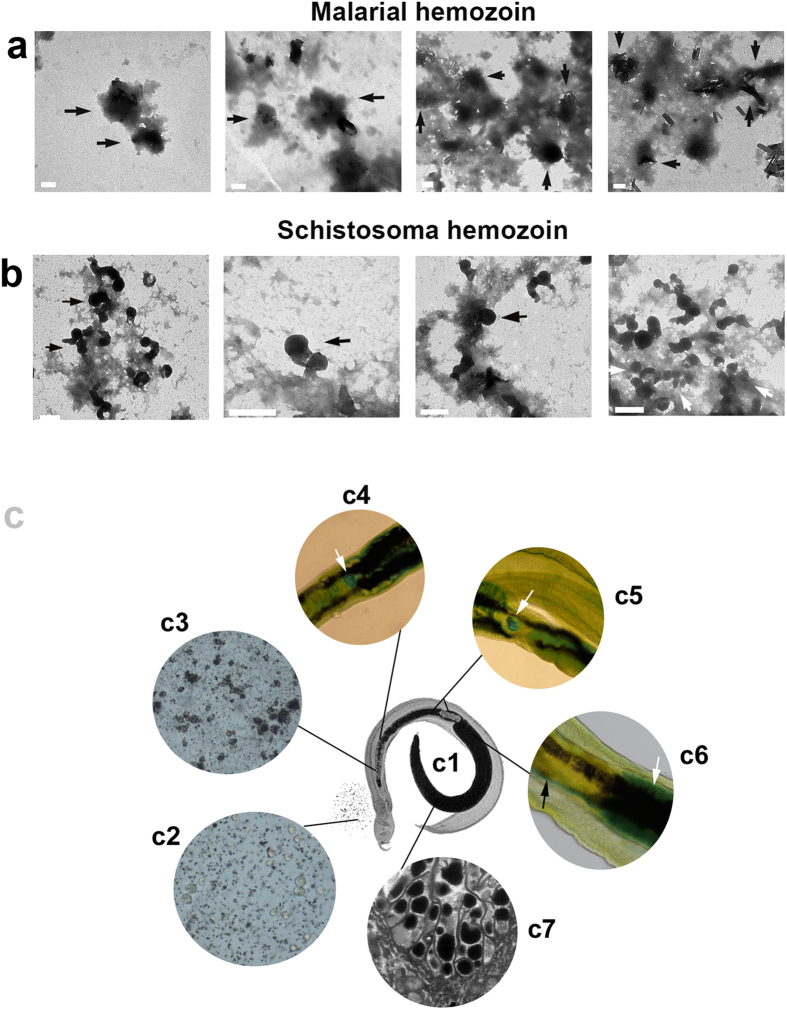
Comparison of morphologies of malarial hemozoin and SHGs and their formation and degradation in *Schistosoma* gut. (**a**) Crystal-like malarial hemozoin formed from the electron-dense part of cell debris. (**b**) Comma-shaped SHGs formed from the electron-dense part of cell debris. All scale bars indicate 1 μm. (**c**) The formation and degradation of SHGs in *Schistosoma* gut. (c1) A pair of *S. japonicum*. (c2) Vomitus from female *S. japonicum* showing the mixture of recently ingested erythrocytes and SHGs. (c3) Erythrocytes were attached to and destroyed by SHGs in the anterior portion of the worm. (c4) *S. japonicum* egg was stained with Prussian blue in the uterus (arrow). (c5) *S. japonicum* egg stained with Prussian blue in ootype(arrow). (c6) The vitelline gland, adjacent to the SHGs-containing posterior portion of the *S. japonicum* gut, was stained with Prussian blue (white arrow). Vitelline gland cells were stained in vitelline duct (black arrow). (c7) Electron dense SHGs were degraded between intestinal villi.

In malarial parasites, we found that, as in SHGs, hemozoin crystal also form from the electron-dense part of cell debris (Fig. 4a), indicating that hemozoin formation depends on the electron-dense part of cell debris. Notably, the electron-dense part of cell debris formed after the cell was attached and degraded by SHGs. It was derived from the cell, and then transformed to a specific form; only then did the hemozoin microcrystal begin to aggregate to form new hemozoin. We observed a clear difference between malarial hemozoin and SHGs in shape (Fig. 4a,b). Such difference likely arises from the different micro-environment in which hemozoin forms. The former is limited to the food vacuole in a cell and maintain a relatively stable state, like a store, whereas the latter forms in *Schistosoma* guts and enables attachment to erythrocytes. Supplementary Fig. 5 shows that the comma-shaped granules easily attach to various cells, suggesting a specific significance of a comma-shaped granule. In addition, aside from hemozoin crystal-like shape, other different shapes of malarial hemozoin are also observed (Fig. 4a and Supplementary Fig. 6). Thus, it is possible that at different developmental stages of malarial parasites, these hemozoin show different morphologies. Although differences in morphology are observed between SHGs and malarial hemozoin as well, both of them are still iron-containing crystals of heme.

In our *in vitro* assay, SHGs did not occur spontaneously in erythrocytes or its debris, even if they contained lipids and heme. However, in the mixture of SGHs and erythrocytes, they can be utilized to form new SHGs. Obviously, erythrocytes plus SHGs meet the recognized requirements of hemozoin formation, including the following: (1) lipids^21^, which are likely from the cell membrane^22^; (2) heme, which come from haemoglobin in erythrocytes; and (3) pre-existing SHGs, which provide seed to initiate the formation of new SHGs^20^. Notably, no enzymes or proteins were added to the culture, suggesting that no proteins or enzymes were necessary during the process, as in malarial hemozoin^20^.

### Interaction between SHGs and other cells aside from erythrocytes

Given that the formation of SHG only requires lipids, heme and pre-existing SHG as seed, we wondered whether cells, aside from erythrocytes, together with heme and SHGs, would likewise promote SHG formation. We found that SHGs attached to and degraded a number of non-erythrocyte cells in the *Schistosoma* gut, similar to the fate of erythrocytes attached by SHGs (Fig. 3k,l). To confirm this finding, we mixed SHGs with erythrocytes (or supernatant of broken erythrocytes by repeated freezing and thawing) and *Saccharomyces* cells *in vitro*. When the mixtures of *Saccharomyces* cells, SHG and erythrocyte supernatant were incubated, dark brown SHG also attached to *Saccharomyces*, thereby leading to the degradation of *Saccharomyces* cells and formation of more SHG (Fig. 3m–p and Supplementary Fig. 7a–d). Upon analysing the amount of SHG in the culture of *Saccharomyces* cells, SHG and erythrocytes, we found that the amount of SHG increased significantly from 1.3 nmol to 11.9 nmol and 37.5 nmol after 4 and 8 days of incubation, respectively (Fig. 3r). This further revealed that other cells, likely acting as another lipid resource, could also promote SHG formation in the presence of heme and SHGs. Furthermore, it clearly showed that different cells (lipids) could lead to the formation of different SHGs with different sizes and morphologies (Supplementary Fig. 7). It also suggests that the morphology of hemozoin is determined by lipid resources or cell environments/conditions. Undoubtedly, in the food vacuole of malarial parasites, the hemozoin and the ingested erythrocyte debris constitute an appropriate system to produce hemozoin. Moreover, its special cell environments/conditions likely result in the special morphology of hemozoin.

The abovementioned results clearly showed the relationship or interaction between erythrocytes (or its debris) and hemozoin (crystal or granules), which further elucidated the model of hemozoin formation. Moreover, SHGs and malarial hemozoin require the same conditions for formation, such as heme and lipids and pre-existing hemozoin. Their similarity suggests that they have the same function. Notably, with the formation of hemozoin, iron (or heme) is undoubtedly transferred from erythrocytes to hemozoin. Moreover, the transfer is performed by a series of “organism-like” behaviors of SHGs, such as attachment to cells, cell lysis and self-assembly.

Unexpectedly, we found that hemozoin granules, which are formed in the anterior portion of *Schistosoma* gut (Fig. 4c3), are degraded again between microvilli in the posterior portion of the gut (Fig. 4c7 and Supplementary Fig. 8), as has been reported in our previous research^19^. Iron was detected in the vitelline gland, vitelline duct, ootype and eggs of female worms (Fig. 4c4–c6). In our previous study, iron was also found in the *Schistosoma* gut wall of male and female worms^19^. This observation strongly suggested that the worms need to obtain iron through the formation and degradation of SHG for nutrition and egg production. That is to say, iron is so essential for schistosomes that they have to adopt the unique way of collecting, transporting and utilizing it. It is not clear why the parasites transfer and utilize iron using hemozoin, but it is likely that when erythrocytes are degraded and utilized to form iron-containing heme crystals *in situ*, few free heme are released to the outside of the cells, as suggested by the colorless supernatant of the Schistosoma gut content after centrifugation. It is clear that iron is required during artemisinin activation and artemisinin is capable of killing schistosomes. Thus, the mechanism of action of artemisinin could involve iron, particularly its utilization in the worm.

The high similarity of malarial hemozoin and SHGs implies that malarial parasites require and utilize iron in hemozoin more than previously expected. It is known that many metabolic processes of the malaria parasite are dependent on iron, such as DNA synthesis, carbohydrate metabolism, proteolysis of host hemoglobin, mitochondrial function and electron transport. However, the significance of iron has not been actually recognized, despite the fact that iron chelators have been found to possess antimalarial activity^23^,^24^. This is because the relationship and interaction between iron utilization and hemozoin formation have not been recognized. Hemozoin has been considered a disposal product of free heme, so its potential function of hemozoin has been neglected. However, based on our observation of formation and degradation of hemozoin in schistosomes, we found that hemozoin is actually a vector or a pool of iron. Formation and degradation of hemozoin serves as a means to store, transport and utilize iron. The growth and development and reproduction of malarial parasites depend so much on iron that iron chelators can kill them. Thus, our findings offer an explanation for why these iron-dependent organisms need to form hemozoin. This is because that hemozoin acts as a safe intermediate to store and transport iron. It is obviously impossible to reach this conclusion simply by observing changes of hemozoin in malarial parasites. However, SHGs in the *Schistosoma* gut offer us a unique chance to understand the process or mechanism.

When artemisinin “consumes” heme^25^,^26^ (or iron^27^) and produces free radicals or heme-artemisinin adducts to inhibit heme crystallization^28^, even accelerating the breakdown of hemozoin^5^, it simultaneously inevitably interferes the normal formation and degradation of hemozoin, uses up iron by forming iron-artemisinin within the parasites, interferes or interrupts the transport and utilization of iron, thereby finally leading to death of the malaria parasites. More importantly, the antimalarial effect of iron chelators highlight the greater need and significance of iron in the survival of malaria parasites, compared with other organisms which are not sensitive to iron chelators. In particular, in hematophagous parasites only malarial parasites and schistosomes are capable of producing hemozoin, suggesting that they have a special dependence on iron. Their special demand for iron likely makes them more susceptible to iron deficiency. Artemisinin and its derivatives can deplete cell iron and kill iron-dependent cells^29^,^30^, and they can also kill ring forms and schistosomula, irrespective of hemozoin, in turn suggesting that malarial parasites and schistosomes are iron-dependent organisms. The parasitized erythrocytes were found to contain more iron than uninfected ones^31^. Moreover, the amount of iron increases as the malaria parasite develops from early ring to late schizont stage^31^. In addition, malarial parasites were reported to require serum iron sources in hosts, even iron deficiency in the host could protest from malaria infection^32^. These works strongly confirmed that malaria parasites actually store, need and depend on iron. For these hemozoin-producing organisms, if hemozoin formation is considered only as the waste of heme detoxification, long-term accumulation of large amounts of hemozoin in cells is difficult to explain. Instead, it is a rational explanation that hemozoin act as an iron reservoir or an iron vector, which continuously provide iron to meet the parasite’s requirement. Indeed, it has been proposed that iron or heme-iron play a pivotal role in the mechanisms of action and toxicity of artemisinin^17^,^25^. Although artemisinin leads to various tissue damages or molecular effects, causing more widespread changes than iron chelators, the ultimate effect of artemisinin or its derivatives is likely on the interference in iron utilization. This hypothesis offers a better explanation for why artemisinin and its derivatives only act on very few types of organisms, such as schistosomes, malarial parasites and other iron-dependent cells, and why a ring form of malaria trophozoite without hemozoin is sensitive to artemisinin. Our hypothesis can reasonably explain most controversial results, whereas other views often cause much controversy. For example, recent proteomics analyses showed that many of the identified artemisinin target proteins were involved in various biological processes, including carboxylic acid metabolic process, cellular biogenic amine metabolic process, nucleoside metabolic process, DNA synthesis, protein synthesis, lipid synthesis, glycolysis and hemoglobin digestion, and so on^25^,^33^. Thus, some researchers even proposed that activated artemisinin kill the malaria parasite through a promiscuous targeting mechanism^25^. If so, it is more difficult to understand the mechanism of action of artemisinin. Undoubtedly, this view does not offer a reasonable explanation for why very limited kinds of organisms can be killed by artemisinin and its derivatives, and for what role the target proteins, such as PfEXP1, PfATP6, PfTCTP, Pfmdr1, K13-propeller protein and PfPI3K, could play independently in the mechanism of action of artemisinin.

Overall, these phenomena, such as the formation and degradation of SHGs in *Schistosoma* gut, transport of iron from guts to eggs, high similarity between SHGs and malarial hemozoin, accumulation of large amounts of malarial hemozoin in food vacuole, the antimalarial effect of iron chelators, and so on, support the rationality of our hypothesis. In addition, it is reasonable that in malarial parasites the alkylation of proteins or damage of cell organs, which are caused by artemisinin and its derivatives, also contribute to parasite killing.

Thus, research on the formation and degradation of SHGs in schistosomes improve our understanding on the significance of malarial hemozoin and the antimalarial mechanism of artemisinin. In particular, the conclusions in this study cannot be drawn simply by researching malarial parasites or the interaction of artemisinin and some proteins or tissue structures in malarial parasites, regardless of the function of hemozoin as iron vector. In addition, our opinion does not exclude previously recognized theories^5^,^6^,^7^,^8^,^9^,^10^,^11^,^12^,^13^, but instead reveals a hidden mechanism. Although our view still needs the support of more evidences, it undoubtedly has provided unprecedented insight into the mechanism of action of artemisinin. Furthermore, our theory also introduces a novel idea for understanding or revealing the mechanism of resistance of artemisinin.

## Methods

### Ethics statement

This study was carried out in strict accordance with the recommendations of the Regulations for the Administration of Affairs Concerning Experimental Animals of the State Science and Technology Commission. The protocol was approved by the Internal Review Board of Tongji University School of Medicine (TJLAC-014–017).

### Parasites and isolation of schistosome hemozoin granules (SHGs)

*Oncomelania hupensis* snails were obtained from the Jiangsu Institute of Schistosome Diseases, Jiangsu province, China. Female mice (Kunming strain) with a weight of 20 to 22 g were purchased from SLRC Laboratory Animal Co., Ltd. (Shanghai, China). All mice were kept under standard acclimatization conditions of 12 h light/dark cycle at 25 °C and water and food were available ad libitum. Cercariae freshly shed by the snails were used to infect mice percutaneously with 40 cercariae each. Adult female schistosomes were recovered by perfusion 42 d to 45 d post-infection, and washed thrice with sterile 0.15 mol/L NaCl solution (normal saline).

SHGs were collected by cutting females into small sections in sterile normal saline solution in eppendorf tubes and collecting the dark suspensions, which were centrifuged at 1,500 × g for 30 s and the worm tissues pellets were discarded. Then, the supernatant was re-centrifuged at 12,000 × g for 10 min. The resulting dark-brown pellets were washed thrice with sterile normal saline and observed or stored at −20 °C before use.

### Observation of SHGs from different sections of the gut in female adult worms

An intact female adult worm was placed on a glass slide, and the spit from the mouth of female worms were collected. The worm was then cut into three sections, and the gut contents from each section were collected for observation using a light microscope (LM, Nikon 50i) and a field-emission gun environmental scanning electron microscope (FEG-ESEM, Quanta 200 F). Some were treated with 1–2.5% SDS for 30–60 min, then rinsed with double distilled water three times, finally dried at room temperature for observation using a transmission electron microscopy (TEM).

### Quantification of SHGs

SHGs obtained from the worm gut or from *in vitro* cultures was washed with 25 mmol/L Tris (pH 7.8) containing 2.5% SDS, followed by centrifugation at 12,000 × g for 10 min. This process was repeated until the supernatant was colorless and without spectrophotometric absorbance at 400 nm. The pellet was dissolved in 250 μL of 2.5% SDS buffer with 20 μL of 2.5 mol/L NaOH. Then, the amount of SHGs was quantified by measuring its absorbance at 400 nm using a Unico spectrophotometer.

### *In vitro* incubation assay

SHGs granules were passed through a filter membrane with 3- or 5-μm pores (Millipore). Then, about 10 nmol or 3 nmol of SHGs was incubated with different cells in 500 μL RPMI-1640 culture system in 1.5 mL Eppendorf tubes for 2 d to 8 d at 37 °C as follows: (1) 100 μL SHGs and 200 μL RPMI-1640 culture medium and 200 μL erythrocytes (final concentration of 2 × 10^3^ to 10 × 10^5 ^per mL); (2) 100 μL SHGs and 200 μL Chloroquine (final concentration 200 to 400 μmol/L) and 200 μL erythrocytes (final concentration of 2 × 10^3^ to 10 × 10^5 ^per mL); (3) 100 μL SHGs and 200 μL erythrocytes (or supernatant of broken erythrocytes by repeated freezing and thawing) and 200 μL *Saccharomyces* cells (final concentration of 0.5 to 5 × 10^3 ^per mL); (4) 300 μL RPMI-1640 culture medium and 200 μL erythrocytes (final concentration of 2 × 10^3^ to 10 × 10^5 ^per mL). All conditions were kept sterile throughout the experiment. The quantity of SHGs was determined by spectrophotometry. Erythrocytes are stained with Giemsa stain (Sigma-Aldrich) according to Giemsa’s Staining Protocol.

### Observation of SHGs granules and malarial hemozoin by transmission electron microscopy (TEM)

Samples of SHGs granules collected from the gut of adult female worms or from the aforementioned cell incubations. Samples of malarial hemozoin collected from the cell culture dish or from liver tissues of infected mice. Every sample was first fixed with 2.5% glutaraldehyde-PBS (phosphate-buffered saline) buffer (pH 7.4) at 4 °C. The samples were then rinsed and stained with or without 1% uranyl acetate for 30 s to 60 s, and dried at room temperature for 5 min. The samples were examined by a JEOL EW-1230 transmission electron microscope at an accelerating voltage of 80 kV, and images were acquired using a digital photo-documentation system (Gatan Bioscan Camera, model 792).

### Observation of SHGs granules by FEG-ESEM

The SHGs specimens collected from the gut of adult female worms or from the aforementioned cell incubations were fixed with 2.5% glutaraldehyde-PBS buffer (pH 7.4) at 4 °C for 1 h to 2 h, then rinsed and fixed with 1% to 4% osmium tetroxide at 4 °C for 1 h to 2 h, and sequentially dehydrated in graded ethanol series. These steps were followed by critical-point drying in CO_2_ and sputter-coating with gold-platinum using standard techniques. The incubated *Saccharomyces* cells and SHGs were directly observed with ESEM (Quanta 200 F) to observe the interaction between SHGs granules and living *Saccharomyces* cells.

FESEM can be used for high-resolution imaging. It can be switched between three vacuum modes, thus enabling the investigation of conductive, non-conductive, and high-vacuum incompatible materials. The high vacuum mode (typically at 10^−5 ^mbar), called the FEG mode in this study, is used for the imaging and microanalysis of conductive and/or conventionally prepared specimens. The low vacuum mode (at <1.3 mbar) is for non-conductive specimens without preparation, whereas the ESEM mode (at <26 mbar) for high-vacuum incompatible specimens. Our conventionally prepared specimens were investigated using the FEG mode, while the *Saccharomyces* cells incubated with SHGs were analyzed using the ESEM mode.

### Prussian blue Iron Stain assay

The Iron Stain Kit is used to identify ferric iron deposits in a female *Schistosoma japonicum*. Prussian blue iron stain assay is performed according to the previous paper^19^.

### Statistical analysis

Results are presented as mean ± standard deviation from at least three independent experiments. Statistical analyses were performed using one-way ANOVA and Student’s t-test. A value of P < 0.05 was considered statistically significant.

## Additional Information

**How to cite this article**: Sun, J. *et al.* Organism-like formation of Schistosoma hemozoin and its function suggest a mechanism for anti-malarial action of artemisinin. *Sci. Rep.* **6**, 34463; doi: 10.1038/srep34463 (2016).

## Supplementary Material

Supplementary Information

Supplementary Video
