# Supplementary material for: Organism-like formation of Schistosoma hemozoin and its function suggest a mechanism for anti-malarial action of artemisinin
Source: Sci Rep. 2016 Oct 3;6:34463. doi: 10.1038/srep34463 (PMC5046088; doi:10.1038/srep34463)
Supplement: Supplementary Information [file srep34463-s1.pdf]

Organism-like formation of Schistosoma hemozoin and its function suggest a mechanism for anti-malarial action of artemisinin

Jun Sun* , Chen Li, Suwen Wang

Institute for Infectious Diseases and Vaccine Development, Tongji University School of Medicine, 1239 Siping Road, Shanghai 200092, PR China

---

*Jun Sun

Siping Rd. No.1239

Tongji University School of Medicine,

Shanghai 200092, People’s Republic of China

E-mail: swksj@tongji.edu.cn

Additional information

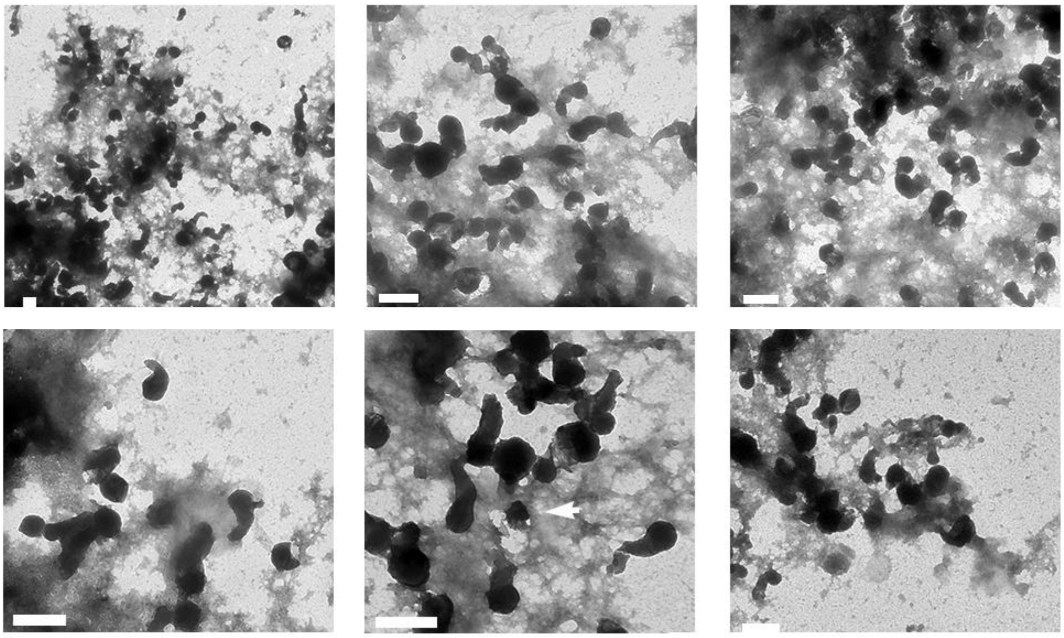

Supplementary Figure 1 | Morphologies of *S. japonicum* hemozoin granules (SHGs). The TEM images showed electron-dense globe- and comma shaped granules. Some granules were forming and attaching to the cell debris. All scale bars indicate 1 μm.

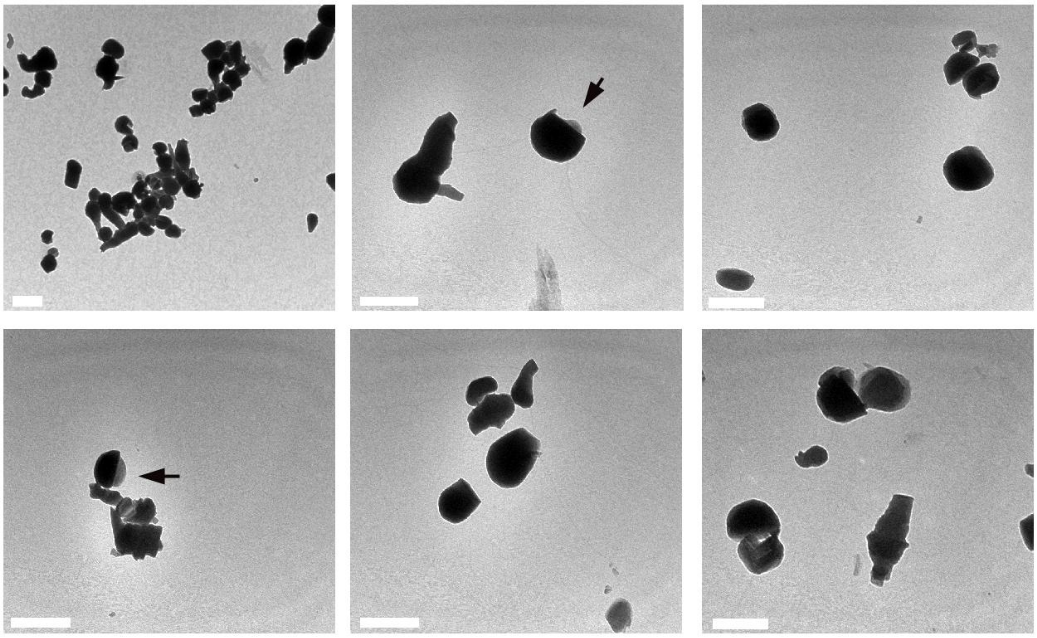

Supplementary Figure 2 丨 Morphologies of *S. japonicum* hemozoin granules after the treatment of SDS solution. After globe- or comma shaped granules were treated with 1% - 2.5% SDS for 0.5-1 hour, TEM images showed that most of the comma-shaped granule tails (arrows) and erythrocyte debris were degraded and the heads were left. All scale bars indicate 0.5 μm.

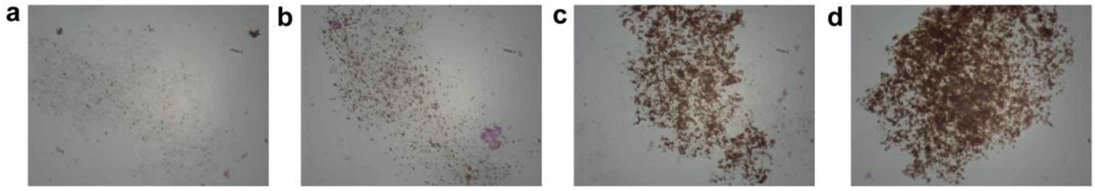

Supplementary Figure 3 丨 A change process of erythrocyte debris after the attachment of a small number of SHGs. (a) A group of erythrocyte debris with a few SHGs in the culture of erythrocytes and individual SHGs. (b- d) The dark brown SHGs gradually formed and the number of SHGs increased gradually with the extended incubation time, without the addition of new SHGs to the culture. All scale bars indicate 5 μm.

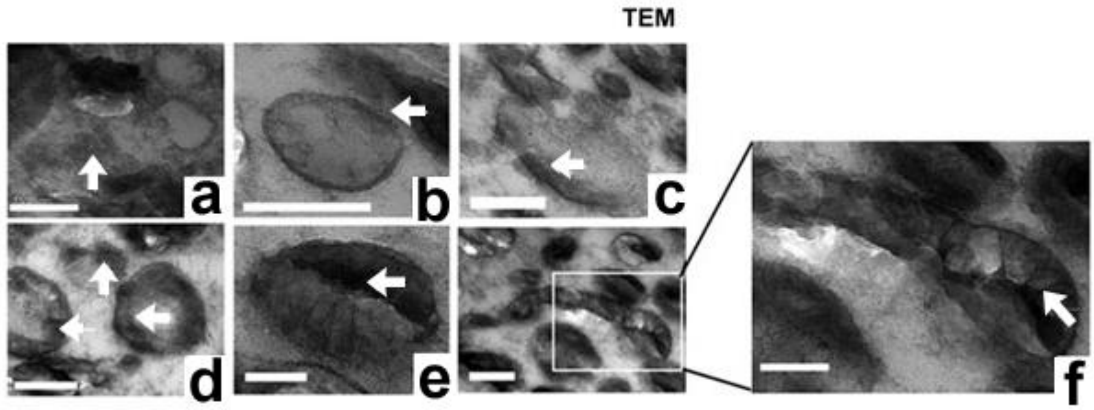

Supplementary Figure 4 丨 The formation process of a comma-shaped granule. (a) The microcrystalline of SHGs (arrow) distributed in the debris. (b) The microcrystalline assembled around the lipid droplets (arrow). (c-d) Microcrystals of SHGs continuously assembled into the polymer (arrow), growing from the edge of the lipid to its core. (e) Microcrystals of SHGs aggregated and formed a globe- shaped granule with a hollow cavity (arrow). (f) Microcrystals aggregated a comma-shaped granule with an unformed tail contacting with the debris. The crystal-like substance was showed in the head (arrow). The scale bar is 200 nm.

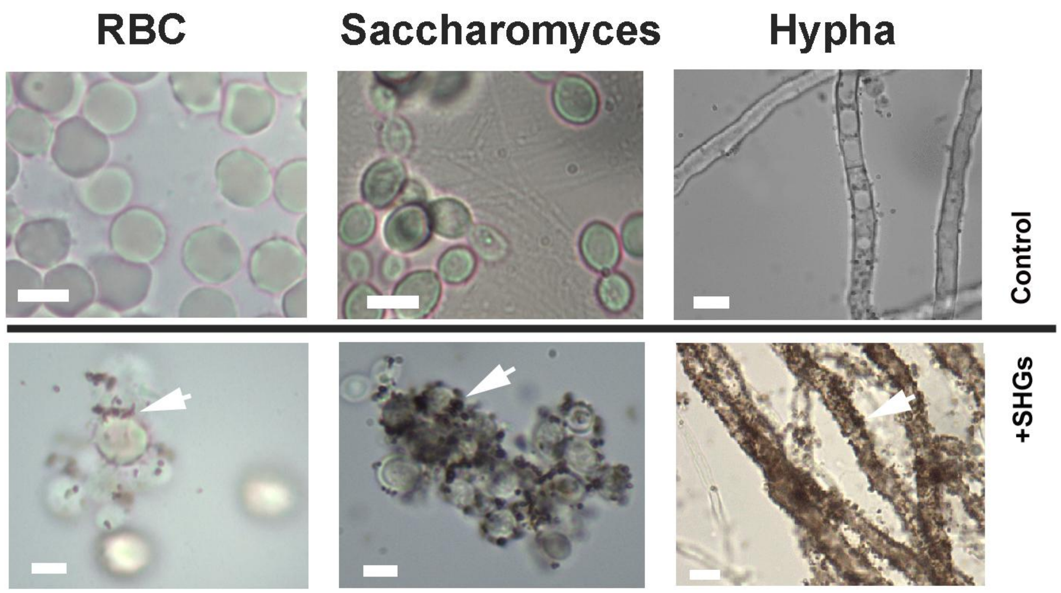

Supplementary Figure 5 | Attachment of *Schistosoma* hemozoin granules to RBC, *Saccharomyces* and Hypha. After SHGs were mixed with RBC, *Saccharomyces* and Hypha, respectively, dark brown SHGs (**arrows**) were capable of attaching to RBC, *Saccharomyces* and Hypha. All scale bars indicate 5 μm.

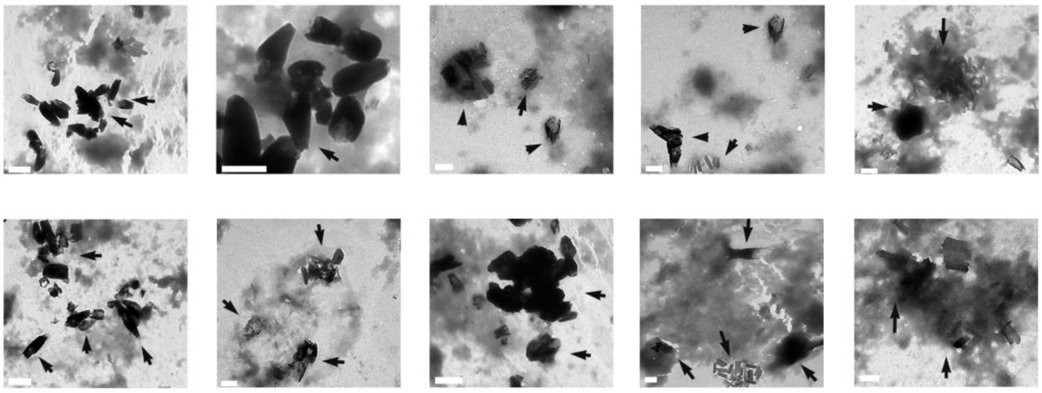

Supplementary Figure 6 | Morphologies of malarial hemozoin granules. The TEM images showed different shapes of hemozoin (arrow). All scale bars indicate 1 μm.

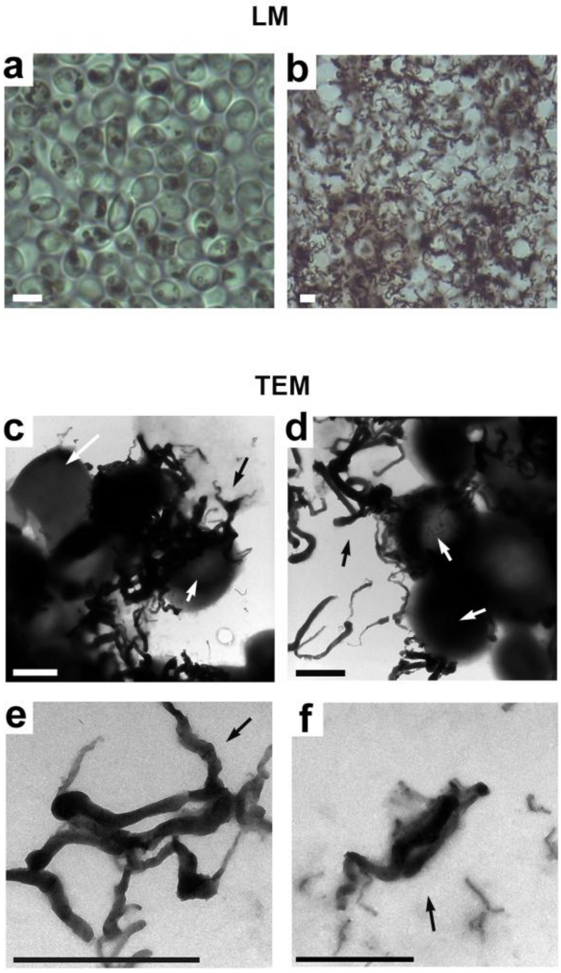

Supplementary Figure 7 | Morphologies and formation of SHGs based the degradation of *Saccharomyces* cells. (a) The LM image showed that in the mixture of *Saccharomyces* cells and SHGs and erythrocyte supernatant, *Saccharomyces* cells were attached by dark brown SHGs. (b) *Saccharomyces* cells were degraded by SHGs, simultaneously dark brown giant SHGs formed, based on the debris. (c-f) These TEM images indicated the giant SHGs (black arrows) and the degraded *Saccharomyces* cells (white arrows). All scale bars indicate 5 μm.

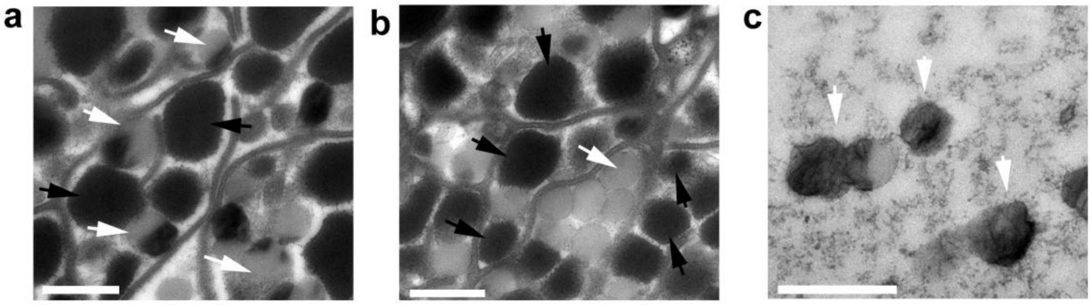

Supplementary Figure 8 | The degraded SHGs between intestinal villi adjacent to the vitelline glands of *S. japonicum*. (a) The TEM image showed the SHGs (black arrows) and degraded SHGs (white arrows). (b) After the SHGs (black arrows) were degraded, lipid drops (white arrows) within the granules were exposed and left. (c) The degraded SHGs (white arrows). All scale bars indicate 0.5 μm.

Video legends

Supplementary files: Video file 1 Formation of *Schistosoma* hemozoin granules. This movie shows a process of *Schistosoma* hemozoin granules (SHGs) formation after erythrocytes are mixed with SHGs, which is similar to what happens in *Schistosoma* gut. The process begins from the attachment of SHGs to erythrocytes, to the degradation of erythrocytes, finally to the formation of clusters of dark brown SHGs. It shows that SHGs utilize erythrocytes to form new SHGs.
